# Association Factors of Self-Management Behaviour Among Lung Transplant Recipients Based on Health Belief Model: A Cross-Sectional Study

**DOI:** 10.3390/healthcare13070782

**Published:** 2025-03-31

**Authors:** Xiaohong Lin, Shaobo Guo, Ruiting Wang, Jiaxin Fang, Xiangru Li, Jing Sun, Yingtian Jia, Hongxia Liu

**Affiliations:** 1 School of Traditional Chinese Medicine, Beijing University of Chinese Medicine, Beijing 100029, China; xiaohong_lin@163.com (X.L.); jiaxin_fang@bucm.edu.cn (J.F.); 2 School of Nursing, Beijing University of Chinese Medicine, Beijing 100029, China; bobo0228guo@bucm.edu.cn (S.G.); 20200935381@bucm.edu.cn (R.W.); lxr2000@bucm.edu.cn (X.L.); 20230935428@bucm.edu.cn (Y.J.); 3 Department of Lung Transplantation, Center of Respiratory Medicine, China-Japan Friendship Hospital, Beijing 100029, China; sj198425@126.com

**Keywords:** lung transplantation, self-management, health belief model

## Abstract

Background/Objectives: Effective self-management after lung transplantation is critical. The Health Belief Model is frequently used to predict and explain the health behaviour in chronic ill patients. The purpose of this study was to explore the status and association factors of self-management among lung transplantation recipients based on the Health Belief Model. Methods: A questionnaire survey was conducted on 123 lung transplantation recipients at the lung transplant unit of a general hospital from March 2022 to October 2023. The survey instruments included the Perceived Social Support Scale, the Champion Health Beliefs Model Scale, and the Self-Management Questionnaire for Lung Transplant Recipients. SPSS 25.0 was adopted to perform descriptive statistics, univariate analysis, and multivariate regression analysis. Results: Scoring indicators of self-management dimensions in lung transplantation recipients were lifestyle management (82.8%), communication with physicians (50.0%), cognitive symptom management (36.0%), and exercise (21.7%). Multiple linear regression analysis showed that the number of postoperative complications, perceived seriousness, perceived benefits, and health motivation explained 22.3% of the total variation in the exercise dimension; health motivation and social support explained 13.5% and 17.4% of the variation in cognitive symptom management dimension and communication with physicians dimension, respectively; and health motivation, social support, and perceived barriers explained 24.0% of the total variation in the lifestyle management dimension. Conclusions: Health motivation, perceived seriousness, perceived benefits and barriers, social support, and the number of postoperative complications were the main association factors of self-management behaviour among lung transplant recipients.

## 1. Introduction

Lung transplantation is an optimal treatment for patients with various end-stage lung diseases. According to International Society for Heart and Lung Transplantation (ISHLT) registration data in 2021, nearly 70,000 adult patients have undergone lung transplantation in the world [1]. Lung transplantation recipients (LTRs) had a median survival of 6.7 years [2]. The Chinese Lung Transplantation Registry System shows that national registered lung transplantation cases reached 2013 by the end of 2020 [3]. In China, the double-lung transplantation survival rates were 78.5% at perioperative period, 64.5% at 1 year, and 48.9% at 3 years; correspondingly, single transplantation survival rates were 83.0%, 69.9%, and 46.8%, respectively [4].

Self-management behaviour plays a critical role in the management of chronic diseases. It refers to the conscious actions and strategies employed by patients to manage their health conditions, including medication adherence, lifestyle modifications, symptom monitoring, and coping strategies. Lung transplant recipients are widely recognized as chronically ill patients [5], who need to adapt to and follow complex self-management tasks to prevent graft rejection, infections, and other complications and maintain graft function as long as possible [6,7]. Specifically, self-management after lung transplantation comprises adherence to a lifelong medical regimen, including medication-taking, self-monitoring of lung function, and recognizing signs and symptoms of complications. Additionally, it involves maintaining a healthy lifestyle such as avoiding harmful substances, attending medical appointments, refraining from smoking, eating well, exercising, and avoiding excessive sun exposure [8].

Self-management is not merely a passive response to illness but an active, ongoing process aimed at improving clinical outcomes and quality of life. Good self-management behaviours are key to maximizing functional recovery, reducing recurrence rates, and long-term survival of LTRs, as well as improving their quality of life [9]. A long-term follow-up study on mHealth interventions demonstrated that enhanced self-management in lung transplant recipients significantly improves clinical outcomes, including reduced mortality and bronchiolitis obliterans syndrome [6]. Some studies have pointed out that LTRs’ inability to establish correct self-management behaviours will directly affect their quality of survival and health status [10]. In addition to clinical benefits, self-management behaviour positively impacts patients’ psychological well-being. By actively engaging in self-management, patients can enhance their sense of control over the disease, reducing feelings of depression [11].

The Health Belief Model (HBM), first proposed by American psychologist Rosenstock in 1966, is one of the most frequently used theoretical model for explaining health-promoting behaviours and guiding the implementation of health interventions [12]. The original HBM suggested that health behaviour facilitators comprise perceived susceptibility (perception of vulnerability to contracting the disease or its consequences), perceived seriousness (perception of the severity or seriousness of the disease or its consequences), perceived benefits (perception of the advantages of engaging in some health behaviour), and perceived barriers (perception of obstacles to engaging in some health behaviour). With the development of the HBM, the concept of self-efficacy and health motivation were added [13,14]. Self-efficacy is the belief that an individual can successfully execute behaviour to achieve desired aims, while health motivation refers to emotional arousal in individuals caused by some health matters.

Perceived susceptibility, perceived seriousness, perceived benefits, perceived barriers, self-efficacy, and health motivation are considered association factors in an individual’s adoption of healthy behaviours and are closely associated with behaviour changes in the HBM. Demographic and sociopsychological variables may affect these above six main variables and finally affect the healthy behaviour change. In addition, this model also proposes that ‘cue to action’, including internal cues (e.g., perception of bodily states) and external cues (e.g., interpersonal interactions), may trigger specific health-related behaviour. Research on health behaviour using the HBM has been applied in diabetic patients’ self-care behaviour, breast cancer screening, COVID-19 vaccination intention, and oral hygiene practice [15,16,17,18].

The health beliefs of patients with chronic respiratory disease such as COPD or asthma have a substantial impact on their health behaviour and effectively predict their self-management behaviour [19,20]. However, limited studies have explored the relationship between the health beliefs of lung transplantation recipients and their self-management behaviour. Therefore, this study investigates the current status of self-management of LTRs and analyse its association factors based on the HBM. The hypothesis of this study is as follows: the self-management behaviour of lung transplantation recipients will be affected by demographic and disease-treatment-related factors, cue to action (social support), perceived susceptibility, seriousness, benefits, barriers, self-efficacy, and health motivation.

## 2. Materials and Methods

### 2.1. Study Design and Participants

This study used a cross-sectional design. From March 2022 to October 2023, a convenience sample of LTRs were selected at the lung transplant unit of a general hospital. Inclusion criteria were as follows: (1) age ≥ 18 years; (2) at least 3 months after lung transplantation; (3) ability to read, understand, and communicate normally; (4) voluntary participation in this study. Exclusion criteria were as follows: (1) having received a multi-visceral or combined organ transplantation; (2) having received second or multiple lung transplantation; (3) having other serious organic diseases. It is commonly recommended that the minimum sample size for a multiple linear regression model should be 5 to 10 times greater than the number of independent variables present in the regression equation [21]. This study involved 21 independent variables; therefore, the appropriate sample size would range from 105 to 210. Consequently, a total of 123 lung transplant recipients were recruited.

### 2.2. Procedures

This study was approved by the Ethics Committee of Beijing University of Chinese Medicine [Approval No. 2022BZYLL0506]. This study was carried out in accordance with the Helsinki Declaration’s ethical principles. Investigators who had received unified training explained the purpose and methods of the study to the participants and assured that withdrawal or declined participation would not affect their further medical service. Participants were also assured that the questionnaires were anonymous, that no personal information would be presented to anyone, and that their responses would be kept confidential. Questionnaires were issued after participants agreed and signed the informed consent form. In order to ensure the authenticity of the data, the recipients filled in the questionnaire by themselves, and the questions they did not understand were explained by the investigators. Most of the recipients agreed to participate in the survey, while a small number of recipients refused to participate due to reasons such as inability to stay seated for a long time, breathing difficulties, and severe hand tremors. Investigators checked and reviewed the filled questionnaire to ensure its consistency and completeness. Data were inputted into Excel sheets by two independent investigators and verified by a third investigator. A questionnaire with 10 consecutive questions on the same option is considered invalid. Missing data were handled by searching electronic medical records or using the mean method.

### 2.3. Measures

#### 2.3.1. General Information Questionnaire

A General Information Questionnaire was used to collect LTRs’ demographic variables and disease-treatment-related variables. Demographic variables included age, gender, residence, employment status, education level, marital status, medical payment method, caregivers, monthly family income, and financial burden. Disease-treatment-related variables included primary disease, length of post-transplant time, type of transplant, and postoperative complications.

#### 2.3.2. Perceived Social Support Scale (PSSS)

A Chinese version of the Perceived Social Support Scale was employed to measure the social support of LTRs. The scale was developed by Zimet [22] in 1988 and was subsequently translated into Chinese and validated among the Chinese population by Huang L and Jiang Q [23] in 1996. The Chinese version scale has been demonstrated to have good reliability and validity by Li L [24] in Chinese LTRs, with the Cronbach’s of 0.85. The scale consisted of 12 items and 2 dimensions: in-family support and extra-family support, using a 7-point scoring method (1 = strongly disagree, 7 = strongly agree) and total score ranging from 12 to 84 points. Scores were summed, and higher scores indicated greater level of perceived social support. In the present study, Cronbach’s α was 0.84.

#### 2.3.3. Champion Health Belief Model Scale (CHBMS)

The Champion Health Belief Model Scale was developed in 1984 by Champion [25], translated into a Chinese version by Wen Z [26], and subsequently revised by Li L and used in LTRs [24], with Cronbach’s ɑ of 0.74. Before the survey, we revised the Chinese version scale and invited five experts to verify the content validity of the scale (CVI = 0.96). The scale contained 36 items and 6 dimensions: perceived susceptibility, perceived seriousness, perceived benefits, perceived barriers, health motivation, and self-efficacy. We replaced “rehabilitation exercise” (in 12 items of perceived benefits and perceived barriers dimensions) with “self-management” and replaced “ventilator limb dysfunction” (in item 2 and 3 of perceived benefits) with “pulmonary dysfunction”. Likert 5-level scoring method was adopted (1 = strongly disagree, 5 = strongly agree). The Cronbach’s ɑ of the scale in this study was 0.82.

#### 2.3.4. Self-Management Questionnaire for Lung Transplant Recipients (SMQLTR)

We modified the Self-Management Questionnaire for Liver Transplantation Recipients, which was initially tailored for liver transplant recipients and demonstrated a Cronbach’s α of 0.87, to better accommodate the specific requirements of lung transplant recipients [27]. The questionnaire comprised 4 dimensions: exercise, cognitive symptom management, communication with physicians, and lifestyle management. We replaced “liver transplant recipients” (item 5 of cognitive symptom management dimension and item 29 of lifestyle management dimension) with “lung transplant recipients”, replaced “water sports” (item 3 of exercise dimension) with “respiratory muscle training”, and added “oxygen saturation” in the self-monitoring item (item 6 of lifestyle management dimension). Exercise was assessed in terms of duration of exercise per week, using a Likert 5-level scoring method (0, no exercise; 1, <30 min per week; 2, 30–59 min per week; 3, 1–3 h per week; 4, >3 h per week). Cognitive symptom management and communication with physicians were assessed using Likert 6-level scoring method (0 = none of the time, 5 = all the time). Lifestyle management included four sub-categories: disease control, diet, sanitation, and activity. The score for each dimension was the mean of its included items, with higher scores indicating better self-management. Scoring indicator of each dimension was calculated (scoring indicator = the median of the dimension/the maximum possible score of the dimension, multiplied by 100.0%), and scoring indicator ≥ 80.0%, 60.0–80.0%, and <60.0% represented a high level, medium level, and low level of self-management, respectively. The standardized Cronbach’s ɑ of the scale in this study was 0.84.

### 2.4. Data Analysis

Data were analysed using SPSS 25.0 software (SPSS Inc., IBM, Chicago, IL, USA). Categorical variables were described by frequencies and percentages. Continuous variables which did not conform to a normal distribution, such as scores of CHBMS, PSSS, and SMQLTR, were described by median, maximum, minimum, and inter-quartile range. Univariate analysis was performed using non-parametric test, Chi-square test, and Spearman’s rank correlation. For multivariate analysis, the score of each dimension in CHBMS was used as the dependent variable, while statistically significant variables in univariate analysis were considered independent variables and then incorporated into the multivariate linear regression equation. The significance level was set at *p* < 0.05 (two tails).

## 3. Results

### 3.1. Descriptive Statistics of Study Participants

A total of 126 questionnaires were collected in this study, and 123 valid questionnaires were retained, with an effective questionnaire rate of 97.6%. The majority of the participants were male. The age distribution was as follows: 11.4% were aged 18 to 44, 30.9% were aged 45 to 59, and 57.7% were aged ≥60. Regarding the duration post-transplant, 27.6% of the participants were at ≤6 months, 18.7% were at 6 to 12 months, and 53.7% were at >12 months. In terms of complications, 59.4% of the participants experienced >3 complications, 39.8% had 1 to 3 complications, and 0.08% did not encounter any complications. Other detailed characteristics, CHBMS scores, and PSSS scores are shown in Table 1. Except for the dimension scores of extra-family support, the remaining scores did not demonstrate a normal distribution.

### 3.2. Component Distribution of Self-Management Behaviour Scores

The component distribution of self-management behaviour scores is shown in Table 2. Among the four dimensions, only the scores of lifestyle management dimension and diet sub-category were normally distributed. As indicated by the scoring indicator, the four dimensions of self-management ranked from high to low as follows: lifestyle management (82.8%), communication with physicians (50.0%), cognitive symptom management (36.0%), and exercise (21.7%). The majority of participants exhibited low levels of scoring indicator in dimensions of exercise, cognitive symptom management, and communication with physicians. Conversely, they demonstrated a high level of scoring indicator in lifestyle management dimension.

### 3.3. Univariate Analysis of Factors Associated with Self-Management Behaviour

The results showed that LTRs’ scores in the exercise dimension had a significant correlation with their number of postoperative complications, perceived seriousness, perceived benefits, health motivation, and self-efficacy. LTRs’ scores in the cognitive symptom management dimension had a significant positive correlation with perceived benefits, health motivation, self-efficacy, and social support. LTRs’ scores in the communication with physicians dimension had a significant positive correlation with perceived benefits, health motivation, self-efficacy, and social support. LTRs’ scores in the lifestyle management dimension had a significant positive correlation with perceived benefits, perceived barriers, health motivation, self-efficacy, and social support (see Table 3).

### 3.4. Multivariate Analysis of Factors Associated with Self-Management Behaviour

The study employed multiple linear regression analysis and backward elimination method to identify the variables. The variables with statistical significance in the univariate analysis were taken as independent variables and four dimensions of SMQLTR as dependent variables. The results showed that LTRs’ number of postoperative complications, perceived seriousness, perceived benefits, and health motivation entered the regression equation for exercise, explaining 22.3% of the variance in the exercise dimension of SMQLTR. Health motivation and social support entered the regression equation for cognitive symptom management and communication with physicians, explaining 13.5% and 17.4% of the variance in these two dimensions of SMQLTR, respectively. Health motivation, social support, and perceived barriers entered the regression equation for lifestyle management, explaining 24.0% of the variance in the lifestyle management dimension of SMQLTR (see Table 4).

## 4. Discussion

The results showed that LTRs displayed low scores in the domains of exercise, cognitive symptom management, and communication with physicians. In contrast, they exhibited a high level of scoring indicator in the dimension of lifestyle management, particularly in the sub-categories of sanitation and disease control. This distinction may stem from the fact that LTRs differ from other chronic disease populations; following their transplant, they are required to take immunosuppressants for life to prevent rejection. The use of these medications can diminish their immune function and resistance, thereby placing them at a heightened risk for infections. The majority of LTRs are aware of their increased susceptibility, recognizing that the occurrence of infection or rejection can increase the risk of poor outcome [28]. This perceived susceptibility likely influences their approach to lifestyle management, prompting them to implement more stringent infection prevention measures than the general population. They also tend to monitor their health more vigilantly and concentrate on adopting healthier lifestyle habits to bolster their immune defences.

In this study, the dimension of exercise exhibited low scores, which is similar to Li’s results [24]. Long-term bed rest pre- and post-transplantation, long-stay hospitalization for acute rejection or infection, and using immunosuppression drugs lead to changes in skeletal muscle groups, which in turn restrict LTRs’ physical activity and exercise tolerance [29]. In addition to physical limitations, factors that prevent lung transplant recipients from exercising include psychological, cognitive, and social factors such as lack of exercise confidence and exercise-related knowledge, discomfort experiences associated with exercise, negative life events after transplantation, concerns about transplant outcomes and health status, and conflicting or ambiguous recommendations from healthcare providers [30]. There are several problems pertaining to LTRs’ exercise status. Primarily, the form of exercise training is single, typically walking, with lack of comprehensive movement planning and strength training. Additionally, LTRs hold excessively high expectations for the outcomes of their exercise endeavours, resulting in disappointment and subsequent reluctance to persist with exercise. Previous study has indicated that exercise training can increase LTRs’ muscle strength and bone mineral density [31]. It is necessary to establish individualized exercise training program in LTRs to enhance their mobility.

The dimension of communication with physicians had a low score in this study. This is likely due to several factors. Firstly, LTRs may not receive the same level of intensive and direct medical care, including frequent communication with physicians, after discharge as they did during their hospitalization. The loss of this direct healthcare support system could potentially impact their communication with healthcare professionals. Secondly, to prevent infections, post-discharge follow-up may rely more on telephone and internet-based interactions rather than face-to-face meetings, which could reduce both the frequency and quality of communication. Furthermore, the shortage of lung transplant specialists and the limited number of beds hinder LTRs from promptly receiving medical consultation, since the lung transplantation field is still in a developmental stage in China. Additionally, in the early period of data collection, the COVID-19 pandemic travel restrictions discouraged movement and thus may have decreased medical visits. While entering the post-COVID-19 era, telemedicine-based strategies may provide an effective approach for facilitating the communication between LTRs and physicians by teleconsultation [32]. The low scores of cognitive symptom management in this study demonstrated that recipients cannot carry out good self-psychological adjust when they experience physical and psychological discomfort. LTRs have significant psychosocial stressors due to the unique transplantation experience and postoperative complications, and post-transplantation psychological stability affects their medical outcomes [33]. Healthcare professionals should pay attention to LTRs’ psychological condition and provide mental health services at the time of follow-up.

An individual’s health motivation is a fundamental prerequisite for their perception and subsequent action. It encourages individual to adopt healthy behaviour based on personal interest and enjoyment, seeking approval from others, enhancing self-esteem, or responding to external pressures [34]. In this study, health motivation was a significant association factor of four dimensions of self-management. A study regarding perceptions and attitudes on mobile health apps in China also found that health motivation could predict health behaviour [35]. To enhance both intrinsic and extrinsic motivation of LTRs, interventions such as health education, peer support, and psychological counselling can be employed.

This study found that social support was an important factor affecting LTRs’ cognitive symptom management, lifestyle management, and communication with physicians. Previous studies have found that lower perceived social support was significantly correlated with unmet care needs of LTRs at home [36]. High social support of LTRs, such as helping, supervising, and reminding from family members, contributed to better adherence to medication, exercise, self-monitoring, and lifestyle.

Our study also found that LTRs who had fewer postoperative complications and higher perceived seriousness or benefits had higher scores in the exercise dimension. Complications after lung transplantation, such as graft dysfunction, rejection, infection, and airway complications, seriously affect LTRs’ physiological function, psychological function, and long-term survival rate. When LTRs realize that poor self-management might lead to serious consequences of their fitness, while adequate self-management can result in favorable outcomes such as reduced complications, diminished symptoms, and enhanced quality of life, they may be motivated to change their unhealthy behaviour and exhibit improved adherence to exercise [24].

The findings of this study indicated that the more barriers LTRs perceived, the lower their lifestyle management scores were. A study of hypertension patients and their caregivers revealed that the perceived barriers influencing their effective self-management consisted of personal factors, family/societal factors, and clinic/organization factors [37]. A multifaceted approach is required to address these obstacles and barriers.

In this study, self-efficacy was positively correlated with all four dimensions of self-management in the univariate analysis; however, it did not remain a statistically significant factor in the multivariate analysis. Similar results were found in a study of adults with multiple chronic diseases [38]. One possible explanation is the influence of multicollinearity, but the correlation between self-efficacy and health motivation (r_s_ = 0.60) was moderate, and the variance inflation factor (VIF) value is less than 5, suggesting that multicollinearity alone may not fully account for this result. The self-management behaviour of LTRs is not solely determined by self-efficacy but may be strongly influenced by environmental factors such as the number of postoperative complications and social support. In particular, even if self-efficacy is high, physical limitations due to postoperative conditions and restricted access to healthcare resources may hinder improvements in self-management behaviour. Additionally, the impact of self-efficacy may be more pronounced in personally adjustable aspects of self-management, such as lifestyle management and cognitive symptom management, while it may be more limited in dimensions dependent on environmental factors, such as exercise and communication with physicians. Therefore, when supporting self-management in LTRs, it is necessary to consider not only enhancing self-efficacy but also addressing physical limitations and strengthening social support to ensure more effective interventions.

### Limitations

This study has some limitations. First, the study was a single-centre study and recruited LTRs at one lung transplant unit in China. The study findings may not be generalized to other geographic regions. Second, the results were based on self-reported questionnaires; therefore, recall bias could be present. Additionally, the sample was represented mainly by men, which may limit the generalizability of the findings, although this is consistent with the overall demographic of lung transplant recipients in China, where males account for approximately 79.6% of the recipients. To address these limitations, large-sample, multi-centre investigations should be performed to provide more potent statistical power. Moreover, more qualitative and mixed-method research could be conducted to further explore these aspects. Furthermore, the results of this study demonstrate that while certain HBM components (e.g., health motivation, perceived benefits, and perceived barriers) are relevant to self-management behaviours in LTRs, other critical factors—such as the number of postoperative complications and social support—also play a significant role. Since social support strongly influences multiple dimensions of self-management (communication with physicians, cognitive symptom management, and lifestyle management), an integrated application of Social Support Theory and Self-Efficacy Theory may provide a more comprehensive understanding. Thus, when designing interventions to promote self-management behaviours in LTRs, it is crucial not to rely solely on the HBM but to incorporate a multifaceted approach that accounts for social support and environmental factors. Future research should combine different behavioural science models to examine the mechanisms of self-management behaviours in greater depth.

## 5. Conclusions

The results of this study revealed that LTRs tended to be mindful of their health and adopt a healthy lifestyle, yet they faced barriers in three key aspects of self-management: exercise, cognitive symptom management, and communication with physicians. Furthermore, health motivation, perceived seriousness, perceived benefits and barriers, social support, and the number of postoperative complications were identified as key factors influencing self-management behaviour. Based on these findings, it is crucial for healthcare professionals to focus on these factors when designing interventions to strengthen self-management among LTRs.

## Figures and Tables

**Table 1:** Demographics, disease-treatment-related profiles, and CHBMS and PSSS scores of LRTs.

Variable	Category	*n* (%)	Min	Max	Median (P25, P75)/Mean ± SD
Age			20	78	62 (54, 68)
Gender	Men	103 (83.7)			
	Women	20 (16.3)			
Residence	Urban	105 (85.4)			
	Rural	18 (14.6)			
Education level	Less than junior college	72 (58.5)			
	Junior college or above	51 (41.5)			
Marital status	Unmarried	5 (4.1)			
	Married	118 (95.9)			
Have caregivers	Yes	105 (85.4)			
	No	18 (14.6)			
Employment status	Employed	16 (13.0)			
	Unemployed or retired	107 (87.0)			
Medical payment method	Out-of-pocket paid	4 (3.3)			
	Medical insurance paid	119 (96.7)			
Monthly family income (CNY)	≤3000	25 (20.3)			
	3000–6000	30 (24.4)			
	6000–10,000	37 (30.1)			
	≥10,000	31 (25.2)			
Financial burden	None	37 (30.1)			
	Mild	22 (17.9)			
	Moderate	24 (19.5)			
	Severe	40 (32.5)			
Type of transplant	Single-lung transplantation	51 (41.5)			
	Double-lung transplantation	72 (58.5)			
Primary disease	Interstitial lung disease	92 (74.8)			
	Chronic obstructive pulmonary disease	9 (7.3)			
	Others	22 (17.9)			
Length of post-transplant time (month)			2	84	15 (6,36)
Number of postoperative complications			0	10	4 (3, 5)
CHBMS total score			86	158	123 (117, 132)
Perceived susceptibility			5	25	15 (11, 18)
Perceived seriousness			7	34	20 (18, 22)
Perceived benefits			10	30	24 (23, 26)
Perceived barriers			6	30	15 (14, 18)
Health motivation			19	35	28 (27, 32)
Self-efficacy			10	25	20 (19, 22)
PSSS total score			35	84	64 (57, 71)
In-family support			12	28	25 (23, 28)
Extra-family support			8	56	37.87 ± 9.75

Note. Abbreviations: CHBMS = Champion Health Belief Model Scale; PSSS = Perceived Social Support Scale; LRTs = lung transplantation recipients.

**Table 2:** Component distribution of SMQLTR scores of LTRs.

Variables	Max	Min	Median (P25, P75)/Mean ± SD	Scoring Indicator	Low Level (*n*, %)	Medium Level (*n*, %)	High Level (*n*, %)
Exercise (min/week)	720	0	195 (180, 300)	21.7%	119 (96.7%)	1 (0.8%)	3 (2.5%)
Cognitive symptom management	5.00	0	1.80 (1.20, 2.60)	36.0%	104 (84.5%)	14 (11.4%)	5 (4.1%)
Communication with physicians	5.00	0	2.50 (2.00, 2.75)	50.0%	93 (75.6%)	24 (19.5%)	6 (4.9%)
Lifestyle management	5.00	2.93	4.14 ± 0.41	82.8%	1 (0.8%)	39 (31.7%)	83 (67.5%)
Activity	5.00	2.00	3.60 (3.00, 4.20)	72.0%			
Diet	5.00	2.63	4.09 ± 0.56	81.8%			
Disease control	5.00	3.13	4.38 (4.00, 4.75)	87.6%			
Sanitation	5.00	2.63	4.38 (4.00, 4.88)	87.6%			

Note. Abbreviations: SMQLTR = Self-Management Questionnaire for Lung Transplant Recipients; LRTs = lung transplantation recipients.

**Table 3:** Univariate analysis of factors associated with self-management behaviour.

Variable	Exercise		Cognitive Symptom Management		Communication with Physicians		Lifestyle Management	
	F/rs/c2	*p*	F/rs/c2	*p*	F/rs/c2	*p*	F/rs/c2	*p*
Age	−0.05 a	0.619	−0.13 a	0.148	−0.03 a	0.716	0.03 a	0.761
Gender	−1.30 b	0.193	−0.26 b	0.794	−1.19 b	0.233	−1.30 b	0.194
Residence	−0.19 b	0.852	−1.58 b	0.113	−1.75 b	0.080	−1.36 b	0.174
Education level	−0.16 b	0.871	0.25 b	0.807	−1.95 b	0.051	−1.26 b	0.209
Marital status	−2.00 b	0.045	−1.63 b	0.104	−1.07 b	0.286	−1.48 b	0.139
Have caregivers	−0.85 b	0.394	−0.37 b	0.714	−0.69 b	0.491	−0.28 b	0.777
Employment status	−0.84 b	0.399	−0.81 b	0.417	−0.08 b	0.937	−0.19 b	0.851
Medical payment method	−1.00 b	0.315	−1.38 b	0.167	−0.03 b	0.977	−1.66 b	0.098
Monthly family income (CNY)	0.30 c	0.961	5.47 c	0.141	3.89 c	0.274	5.02 c	0.170
Financial burden	4.30 c	0.231	1.99 c	0.575	2.60 c	0.457	1.45 c	0.695
Type of transplant	−1.16 b	0.247	−0.25 b	0.803	−1.86 b	0.063	−0.41 b	0.679
Primary disease	4.81 c	0.090	0.20 c	0.906	0.36 c	0.834	3.48 c	0.175
Length of post-transplant time (month)	0.11 a	0.208	0.06 a	0.512	−0.18 a	0.052	0.01 a	0.909
Number of postoperative complications	−0.23 a	0.011 *	0.11 a	0.243	0.08 a	0.407	0.01 a	0.887
Perceived susceptibility	−0.15 a	0.095	0.04 a	0.643	0.06 a	0.501	−0.01 a	0.924
Perceived seriousness	−0.24 a	0.007 **	−0.06 a	0.497	−0.10 a	0.288	−0.05 a	0.609
Perceived benefits	0.29 a	0.001 **	0.22 a	0.013 *	0.19 a	0.033 *	0.18 a	0.048 *
Perceived barriers	−0.16 a	0.075	−0.15 a	0.100	−0.07 a	0.455	−0.24 a	0.007 **
Health motivation	0.30 a	0.001 **	0.32 a	<0.001 **	0.36 a	<0.001 **	0.44 a	<0.001 **
Self-efficacy	0.21 a	0.019 *	0.23 a	0.010 **	0.21 a	0.019 *	0.34 a	<0.001 **
Social support	0.08 a	0.367	0.30 a	0.001 **	0.30 a	0.001 **	0.23 a	0.010 *

a rs value, b F value, c c2 value, * *p* < 0.05, ** *p* < 0.01.

**Table 4:** Multivariate analysis of factors associated with self-management behaviour.

Variable	B	SE	β	t	*p*	R2	F
Constant	−35.06	117.42		−0.30	0.766		
Number of postoperative complications	−10.64	5.58	−0.16	−1.91	0.059		
Perceived seriousness	−6.59	2.37	−0.23	−2.78	0.006		
Perceived benefits	7.53	3.76	0.19	2.00	0.047		
Health motivation	8.74	3.60	0.23	2.42	0.017		
Model 1 (Dependent: Exercise)						0.22	8.44 (*p* < 0.001)
Constant	−1.18	0.74		−1.60	0.113		
Health motivation	0.08	0.02	0.28	3.18	0.002		
Social support	0.02	0.01	0.17	1.90	0.060		
Model 2 (Dependent: Cognitive symptom management)						0.14	9.35 (*p* < 0.001)
Constant	−0.71	0.62		−1.15	0.254		
Health motivation	0.08	0.02	0.33	3.85	0.000		
Social support	0.01	0.01	0.17	2.00	0.048		
Model 3 (Dependent: Communication with physicians)						0.17	12.66 (*p* < 0.001)
Constant	2.86	0.33		8.74	<0.001		
Health motivation	0.04	0.01	0.38	4.51	<0.001		
Social support	0.01	0.00	0.14	1.72	0.087		
Perceived barriers	−0.02	0.01	−0.18	−2.29	0.024		
Model 4 (Dependent: Lifestyle management)						0.24	12.52 (*p* < 0.001)

## Data Availability

The datasets used and analysed during the current study are available from the corresponding author on reasonable request.

## Abbreviations

The following abbreviations are used in this manuscript:

LTRs	Lung transplantation recipients
HBM	Health Belief Model
SMQLTR	Self-Management Questionnaire for Lung Transplant Recipients
CHBMS	Champion Health Belief Model Scale
PSSS	Perceived Social Support Scale
